# Two‐Regime Conformation of Grafted Polymer on Nanoparticle Determines Symmetry of Nanoparticle Self‐Assembly

**DOI:** 10.1002/advs.202406720

**Published:** 2024-07-29

**Authors:** Ji Woong Yu, Hongseok Yun, Won Bo Lee, YongJoo Kim

**Affiliations:** ^1^ Center for AI and Natural Sciences Korea Institute for Advanced Study Seoul 02455 Republic of Korea; ^2^ Department of Chemistry and Research Institute for Convergence of Basic Science Hanyang University Seoul 04763 Republic of Korea; ^3^ School of Chemical and Biological Engineering Institute of Chemical Processes Seoul National University Seoul 08826 South Korea; ^4^ School of Transdisciplinary Innovations Seoul National University Seoul 08826 Republic of Korea; ^5^ Department of Materials Science and Engineering Korea University Seoul 02841 Republic of Korea

**Keywords:** grafted nanoparticle, molecular dynamics simulation, nanoparticle self‐assembly, polymer conformation, self‐assembly symmetry transition

## Abstract

One of the key design factors that regulate the properties of grafted nanoparticles (GNPs) and their self‐assembly is the conformation of the grafted polymer. On the curved surface of the GNP core, the conformation of the polymer chain is not uniform in the radial direction. The segment is a non‐Gaussian chain in the concentrated polymer brush (CPB) regime near the interface between GNP core and grafted polymer, while it is less constrained in the semidilute polymer brush (SDPB) regime near the surface of GNP. Here, the property of polymer conformation showing crossover behavior at the CPB/SDPB threshold through the coarse‐grain molecular dynamics simulation of nanoparticles with explicit grafted chains is explored. Moreover, the self‐assembly structure depends on the effective softness, which is defined as a function of the threshold of two regimes estimated from the conformation of the polymer.

## Graphical abstract

The study examines the transition between concentrated polymer brush and semidilute polymer brush regimes through polymer conformation analysis. It precisely determines effective softness as an order parameter predicting self‐assembly symmetry. The research utilizes self‐assembly simulations to demonstrate that the symmetry predicted by effective softness aligns with simulation results. The study provides insights into nanoparticle arrangements based on polymer grafting properties.

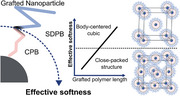

## 1 Introduction

The field of nanoparticle engineering has seen significant progress, beginning with the creation of long‐range ordered structures^[^ ^1^, ^2^, ^3^, ^4^, ^5^ ^]^ and culminating in the recent application of superlattice assembly in various fields such as electronics^[^ ^6^, ^7^ ^]^ photonics,^[^ ^8^, ^9^, ^10^, ^11^ ^]^ and magnetics.^[^ ^12^, ^13^, ^14^ ^]^ One key aspect of this development has been the manipulation of grafted nanoparticle (GNP) design through various methods, including modifying the core of GNPs themselves or using polymeric ligands. Altering the GNP cores involves changing their composition,^[^ ^15^, ^16^ ^]^ size,^[^ ^17^, ^18^ ^]^ and geometry,^[^ ^19^, ^20^, ^21^, ^22^ ^]^ while the use of polymeric ligands involves passivating soft ligands that serve as a modulator for inter‐GNP interaction by adjusting the degree of polymerization or grafting density.^[^ ^23^, ^24^, ^25^, ^26^, ^27^, ^28^, ^29^ ^]^

While the effects of both ligand and GNP core can be examined separately, recent studies have introduced a parameter called “softness” (λ),^[^ ^30^, ^31^, ^32^ ^]^ which considers both effects together in determining the symmetry of GNP superlattices. The softness is defined as the ratio of the stretched length of the grafted ligands (*L*_graft_) to the radius of the GNP core (*R* = 0.5*D*) where *D* is diameter of GNP core. When the softness is below a range of 0.6−0.7, the GNP superlattice exhibits a close‐packed structure with either face‐centered cubic (fcc) or hexagonal close‐packed (hcp) symmetry. Above this range, the structure becomes non‐close‐packed, with either body‐centered cubic (bcc) or body‐centered tetragonal (bct) symmetry. This structural transformation bears a resemblance to that observed in linear diblock copolymers.^[^ ^33^, ^34^, ^35^, ^36^ ^]^ Specifically, in linear diblock copolymers, the minor component (analogous to the GNP core) self‐assembles into isolated bcc‐symmetry domains, while the major component (analogous to the grafted polymer) fills the surrounding space in a continuous manner. The transition between these two symmetries is thought to be related to the balance between the enthalpy advantage of the close‐packed structure and the entropy increase associated with longer ligands in the non‐close‐packed structure.^[^ ^37^ ^]^

**Figure 1:**
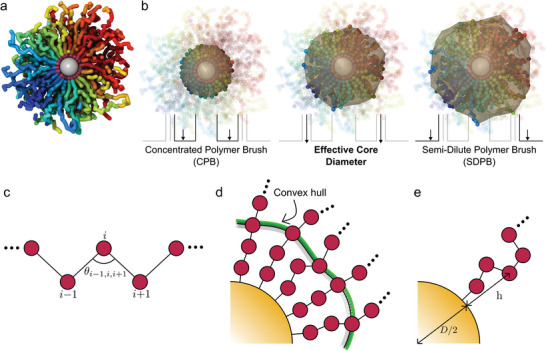
The cross‐section of GNP, CPB, and SDPB, and polymer conformation analyses. a) Cross‐section of GNP (*D* = 4.5σ, *N*_graft_ = 180, *L*_graft_ = 24). b) Two regimes (CPB and SDPB) of GNP corona divided by a boundary at which we can define effective core diameter and corresponding convex hull generated using the alpha‐shape method.^[^ ^46^ ^]^ The schematic diagrams of c) cosine bond angle (CBA), d) convex hull, and e) grafted polymer height are illustrated for better understanding. The definition of each parameter can be found in the Experimental section.

The concept of the “softness” parameter has been further developed to include additional characteristics of GNPs. In the previous study, it was demonstrated that the softness parameter was insufficient in explaining symmetry transitions upon varying grafting density, and the “effective softness” (λ_eff_) was introduced as an alternative.^[^ ^24^, ^25^ ^]^ This modification considers the fact that the contribution to chain packing entropy is lower for ligand monomers near the GNP core surface due to their limited free volume. The nonuniform conformation can also be observed directly through crosssection view of single GNP in **Figure** **1**a. This results in a portion of the ligand behaving more like the GNP core, and the diameter of the effective core diameter (*D*_eff_) is redefined accordingly. The effective softness can then be defined as λ_eff_ = *L*_eff_/*D*_eff_ , where *L*_eff_ is the length of the remaining ligand. The study found strong correspondence between symmetry and λ_eff_, indicating that the symmetry of GNP superlattices cannot be considered independently from the way in which the grafted polymers are structured on the surface of the GNP core.

Theoretical descriptions of the non‐uniform conformation of grafted polymer on the curved surface of GNPs often draw upon the Daoud and Cotton (DC) blob scaling theory of arm in star polymer.^[^ ^38^ ^]^ This is because the small core limit of GNPs resembles a star polymer, while the other end exhibits hard‐sphere behavior. Ohno et al. extended the DC theory to GNP,^[^ ^39^ ^]^ proposing that the polymer brush height (*h*) scales differently in two distinct regimes. In the proximity of the GNP core surface, the grafted polymers are strongly constrained and exhibit non‐gaussian behavior. This regime is known as the concentrated polymer brush (CPB; Figure 1b left), and the grafted polymer heights scale as $h∼N^{4/5}$ (in experiment), where *N* is the number of polymer segment monomers.^[^ ^40^ ^]^ After a theoretically predicted critical height (*r*_c_) from the surface, the heights scale as $h∼N^{3/5}$ in another regime called the semidilute polymer brush (SDPB; Figure 1b right). In this regime, the polymer behaves similarly to a swollen star polymer. The threshold dividing these two regimes corresponds to the point at which the polymer recovers star polymer behavior.

The complexity of corona can be analyzed as an independent characteristic of GNP itself, but the heterogeneity can also be considered through interaction with neighboring GNPs. In the work of Midya et al.,^[^ ^41^ ^]^ they showed a theoretical two‐state model wherein the corona can be divided into an interpenetration layer that overlaps with the corona of neighboring GNPs and a dry layer unperturbed by the neighboring GNPs. The research showed the correspondence between the theoretically predicted transition point between two layers and the prediction based on molecular dynamics simulation in terms of extension free energy. Another notable reference is the scaling theory of the viscoelastic behavior of star polymers, as detailed by Kapnistos et al.,^[^ ^42^ ^]^ which explores the viscoelastic behavior, taking into account the influence of neighboring GNPs.

The existence of multiple heterogeneities in the corona prompts the question of how the *D*_eff_ can be defined given these heterogeneities. The *D*_eff_ is typically known to fall between two different regimes (CPB and SDPB) as shown in Figure 1b but less effort has been made on polymer conformation and its relationship to self‐assembly. To this end, we performed extensive coarse‐grained molecular dynamics simulations on a large number of GNPs with explicitly grafted polymers. Our results are consistent with previous reports by the authors and confirm the reproducibility of the observed symmetry transition with varying *L*_graft_. Notably, this study represents the first reported instance of a self‐assembly symmetry transition from a random initial configuration involving GNPs with explicit grafted polymers which causes the computational cost to skyrocket. A limited number of related studies have examined the symmetries of GNP self‐assemblies in pre‐organized configurations at the unit‐cell or small superlattice level.^[^ ^32^, ^43^, ^44^ ^]^ Subsequently, we investigated the conformational transitions of grafted polymers located at specific distances from the GNP core surface using various metrics shown in Figure 1c–e. Our findings align with the theoretical framework proposed by Ohno et al.^[^ ^39^ ^]^ However, we also observed a continuously changing scaling exponent, corroborating a recent experimental study,^[^ ^45^ ^]^ in contrast to the respective fixed exponents in different regimes. We identified two distinct types of transitions and, by examining the monomer indices associated with these transitions, we were able to obtain a range of effective softness values. By comparing our results with experimentally observed GNP superlattice phase transitions, we have established an appropriate definition of the *D*_eff_, which in turn determines the CPB‐to‐SDPB transition. Our simulation thus offers a novel approach to link the macroscopic behavior of the GNPs to the microscopic properties of the grafted polymer.

## 2 Results

### 2.1 Symmetry of Self‐Assembly Structure

**Figure 2:**
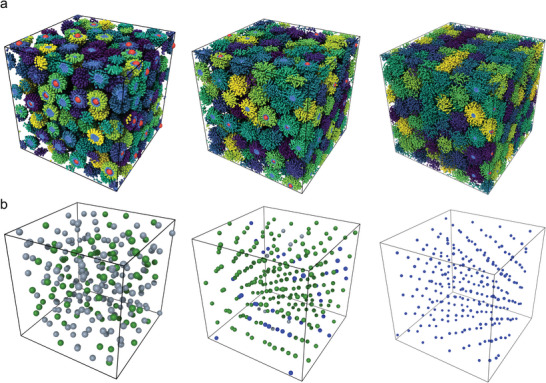
Snapshots of MD simulations of GNPs with different *L*_graft_. MD simulation snapshots of GNPs with varying *L*_graft_ values. From left to right, *L*_graft_ =  4, 10, and 24, at *D* = 3.75σ and *P*_4_ = 10^−2.125^ ε/σ^3^ a) Visualization of grafted monomers (light blue) and GNP cores (red). Colors, excluding red and light blue, are assigned based on the molecular index using the viridis color map, creating distinct colors for each GNP. b) GNP core visualization showing disordered (gray), close‐packed (green), and bcc (blue) structures, classified by VoroTop. Note that single snapshot classifications may differ from time‐averaged statistical classifications.

The first interest of our research is whether it is indeed possible to obtain a self‐assembling structure starting from a random initial configuration. And whether such a self‐assembly structure can show a symmetry transition trend as we have shown in our previously reported experiments.^[^ ^24^, ^25^ ^]^ Thus, in this section, we focus on the macroscopic structure of GNP self‐assembly. Our objective is to identify the parametric window in which different symmetries (non‐close‐packed and close‐packed) emerge. To implement the grafting between the GNP core and the grafted monomer, the GNP core and the grafted monomers form a rigid body. Weeks‐Chandler‐Andersen (WCA) monomers connected by finitely extensible nonlinear elastic (FENE) bonds have been demonstrated to simulate polymers in a good solvent limit (as detailed in the Experimental section). The lack of attractive interactions in the system implies that a certain pressure is necessary to drive self‐assembly. However, determining the appropriate pressure is not straightforward. Experiments on soft colloids^[^ ^47^ ^]^ and theoretical work in the framework of mode coupling theory (MCT) for star polymers^[^ ^48^ ^]^ have shown that above a certain pressure (or density) the system becomes vitrified and enters a non‐ergodic regime. Since that GNPs are unlikely to reach global equilibration within a practical time frame, as previously noted by Midya et al.,^[^ ^41^ ^]^ our observations were made at the boundary of the marginally ergodic state of the system. To avoid any kinetic factors that may disrupt the marginal stability of ergodicity, we performed slow sequential compression from pressure *P*_1_ to *P*_5_ (see Experimental section for iteration scheme). **Figure** **2**a shows an example of the structure of GNPs with different *L*_graft_.

**Figure 3:**
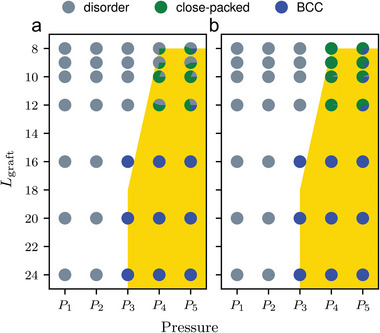
Phase diagram of the self‐assembly of GNPs determined through two distinct methods of characterizing per‐particle local structure. The phases identified using a) i‐CNA and b) VoroTop are illustrated, wherein the area on the face of markers represents the relative fraction of particles with different symmetries. Pressures corresponding to *P*_1_ through *P*_5_ are detailed in the Experimental section. The representation of disordered, close‐packed, and bcc structures is depicted in gray, green, and blue, respectively. It is noteworthy that the close‐packed structure encompasses both fcc and hcp structures. The yellow shaded region represents nonergodic state which is determined through MSD. The diameter (*D*) of the GNPs is held constant at *D* = 3.75σ.

Next, to test whether there is a transition from close‐packed structure to bcc transition as the *L*_graft_ increases at sufficiently high pressure (density), we applied per‐particle symmetry identification methods. This method allows you to determine symmetry at the level of each particle as shown in Figure 2b. To ensure that the transition is not affected by the per‐particle identification method used, two different methods were employed: interval common neighbor analysis (i‐CNA)^[^ ^49^ ^]^ and VoroTop.^[^ ^50^ ^]^ The i‐CNA method is derived from CNA, a parameter‐free structural classification approach, with modifications. On the other hand, VoroTop is a structural classification method based on the topology of Voronoi cells in 3D space. Both methods (**Figure** **3**a,b) confirm the trend of close‐packed to bcc structure with increasing *L*_graft_, indicating the consistency with our previous experiments.^[^ ^24^, ^25^ ^]^ Although the population of each phase showed minor fluctuations due to the lack of convergence to the global equilibrium, the clear emergence of dominant species is observed.

As described above, nonergodicity is expected at high pressure and the rigorous identification of it should be dealt with extensive methods, including MCT and large deviation in independent sampling. However, in practical applications, nonergodicity can be approximated as a significant slowdown (vitrification) of the system dynamics, leading to a lack of sampling the entire phase space and the emergence of inaccessible states within a practical timescale. As a result, in the context of this study, a sampling point is deemed non‐ergodic for practical purposes if the mean square displacement (MSD) of the GNP core within the trajectory at the sampling point is less than 10^2^σ^2^. The non‐ergodic region can be seen in Figure 3a,b as the yellow shaded area.

### 2.2 Conformation of Grafted Polymer

**Figure 4:**
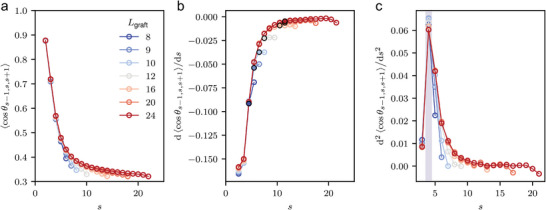
The measured cosine bond angle (CBA) at each monomer, and the first and the second successive derivative of CBA. a) The index assigned to each atom in the system is set up in such a way that the bonded atom directly next to it has a consecutive index number, and the first index is given to the monomer that is attached to the core of the nanoparticle (*s* = 1). b) The first derivative of CBA. The derivative is approximated as a successive differentiation of CBA. The assumed transition points (type II) are denoted by bold black edge and solid face color. c) The second derivative of CBA. The shaded region denotes the assumed transition point (type I) located at the peak of the second derivative. Note that in all cases the last CBA which involves chain‐end monomer is not included. The setup except for the chain length (*L*_graft_) is fixed to *P*_4_ = 10^−2.125^ ε/σ^3^, D = 3.75σ, *N*_graft_ = 125.

Having established that the simulation system utilized in our study can replicate the shift in GNP packing symmetry, our next objective is to elucidate the connection between the conformation of the polymer grafts and the GNP packing symmetry. Firstly, we calculated the cosine bond angle (CBA; see Figure 1c) at each monomer index (*s*), counting from the grafted monomer (*s* = 1), which is a member of the rigid body (see **Figure** **4**a). The CBA is largely related to “local” persistence length.^[^ ^51^, ^52^, ^53^ ^]^ In the theoretical framework^[^ ^39^ ^]^ of Ohno et al., the reported CPB‐to‐SDPB crossover point does not change scale with the chain length, implying the unique *D*_eff_ exists for fixed grafting density. With this prediction, we also found a single uniquely characterized conformation at which the second derivative of CBA peaks (see Figure 4c) and it is expected that a short‐length polymer would exhibit similar conformation to that of a sub‐segment of a long‐grafted polymer chain. However, as depicted in Figure 4a, the CBA curves do not overlap each other. The deviation can be attributed to the presence of open boundary conditions on the free ends of chains. Interestingly, we have identified an additional characteristic length (transition point) that scales with grafted polymer length. We determined this by estimating the deviation of the first derivative of CBA in conformation from an infinitely long grafted chain (or in this case, the longest chain as a substitution) and the point at which deviation begins to increase (see Figure 4b). We designated the transition point that remained invariant of chain length as type I, whereas the one scaling with chain length was classified as type II transition. The criteria employed to identify other types of transition in our research will follow the same definition. In the later part of this work, we will identify which of this is relevant to the symmetry of GNP self‐assembly.

**Figure 5:**
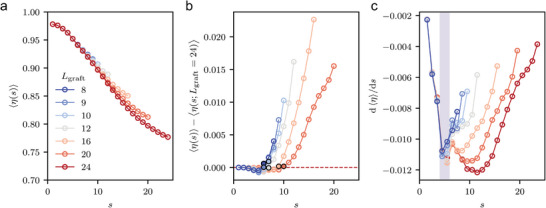
The examination of η for varying *L*_graft_. a) η as a function of *s* for different *L*_graft_. b) The deviation from an infinitely long grafted polymer, with the longest grafted polymer (*L*_graft_ =  24) used as an approximation for infinitely long grafted polymer. Characteristic monomers at which deviation from the longest grafted polymer begins to increase are highlighted with a bold black edge and solid face color. c) The derivative of η, with the region where the first minima occur shaded for emphasis. The setup except for the *L*_graft_ is fixed to *P*_4_ = 10^−2.125^ ε/σ^3^, D = 3.75σ, *N*_graft_ = 125.

As a second approach to characterize conformation, we calculated the sphericity (η(*s*); see the Experimental section for the definition) of the polymer as shown in **Figure** **5**a. The concept behind measuring sphericity is that changes in the surface area and volume enclosed by the 3D configuration of the monomers, up to certain *s*, can provide insight into the polymer's conformation. In the initial rod‐like regime, the convex hull (see Figure 1d) is spherical (see Figure 1b, left), leading to a value of η ≈ 1 for *s* ≈ 1. As more monomers are included (*s* ≫ 1), the convex hull gradually becomes oblique in shape (η < 1) due to the expansion of free volume (see Figure 1b, right). The expanded free volume results in a broader distribution of monomer heights from the GNP surface and a non‐uniform distribution of points on the surface (see Figure 5a). Our findings in the analysis of CBA were consistent with the presence of two distinct characteristic lengths in η. Similar to the case of CBA, the deviation of shorter chains from the longest chain results in a characteristic length that scales with chain length, as depicted in Figure 5b. Type II transition points for each chain length can be extracted from this deviation. Additionally, an examination of the first derivative of η reveals the first minimum at another characteristic length (type I) across all *L*_graft_, as illustrated in Figure 5c. It is noteworthy that although the transition points obtained from η differ numerically from those obtained from CBA, our analysis still revealed two qualitatively different types of transitions.

**Figure 6:**
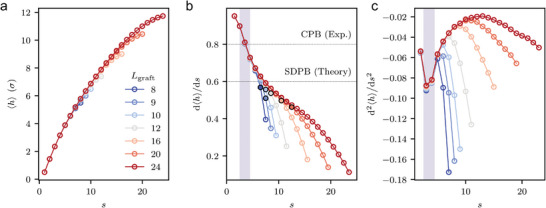
The examination of 〈*h*〉 with *L*_graft_. a) 〈*h*〉 as a function of *s* for different chain lengths. b) The first derivative of 〈*h*〉 with respect to its monomer index. Characteristic monomers at which deviation from the longest grafted polymer begins to increase are highlighted with a bold black edge and solid face color. c) The second derivative of 〈*h*〉 with respect to monomer index, with the region where the first minima occur shaded for emphasis. The setup except for *L*_graft_ is fixed to *P*_4_ = 10^−2.125^ ε/σ^3^, *D* = 3.75σ, and *N*_graft_ = 125.

Lastly, we analyzed the height of the grafted polymer (see Figure 1e) from the surface of the GNP core (〈*h*〉; see **Figure** **6**a). Similar to earlier two results of conformation analyses, our observations on 〈*h*〉 revealed two distinct transitions. The first transition was found near the surface of the GNP core (see Figure 6c) and the second transition was found to be proportional to the length of the grafted polymer and relatively further from the surface (see Figure 6b). The transition point in the first case (type I) was determined as the point at which *d* ^2^〈*h*〉/*ds* ^2^ reached its minimum. On the other hand, the transition points in the second case (type II) were extracted by calculating the deviation of the first derivative as the longest chain began to grow for each *L*_graft_. Although the scaling is a function of the *L*_graft_, the behavior of 〈*h*〉 as a function of *s* was found to almost collapse onto each other, as observed in Figure 6a. For ease of analysis, we investigated 〈*h*〉(*s*) instead of 〈*h*〉(*L*_graft_). The scaling of 〈*h*〉 as a function of *L*_graft_ is presented in Figure S1 (Supporting Information). Contrary to the conventional notion that the scaling exponent ν, $\left\langle h\right\rangle ∼s^{\nu }$, would be 3/5<ν≤1 for CPB and ν  =  3/5 for SDPB, our results indicate otherwise as shown in Figure 6b. While the experiment by Dukes et al.^[^ ^40^ ^]^ reported an exponent of ν = 4/5, the overall successive derivative of 〈*h*〉 at each monomer index (*s*) showed a continuously changing behavior aligning with an experimental report by Hore et al.,^[^ ^45^ ^]^ ranging from 1.0 (rod‐like polymer) to a value less than 0.5. We found a correspondence between the type I transition and the experimental exponent ν = 4/5. Specifically, the monomer index where the type I transition occurs ($s_{c,I}^{\left\langle h\right\rangle }=3.5$) corresponds to a point where the first derivative of the height profile with respect to the distance from the substrate equals 4/5, implying a potential correlation with experimentally determined exponents.

So far, we have conducted a thorough examination of three distinct analyses that characterize the conformations of grafted polymers (CBA, η, and 〈*h*〉). In each analysis, we have identified two qualitatively distinct types of transitions (type I and II) that result in varying values of the monomer index, denoted as $s_{c,\alpha }^{\beta }$ where α=I and II, and β=CBA, η, and ⟨h⟩, at which the transitions occur. Moving forward, we aim to explore the connection between the effective softness assessed from the different transition points and the symmetries of the GNP superlattice. Specifically, we seek to establish how the softness of the grafted polymer layers at these transition points relates to the underlying symmetries of the GNP superlattice.

## 3 Discussion

### 3.1 Link between Grafted Polymer and Superlattice Symmetry: Revising Effective Softness

**Figure 7:**
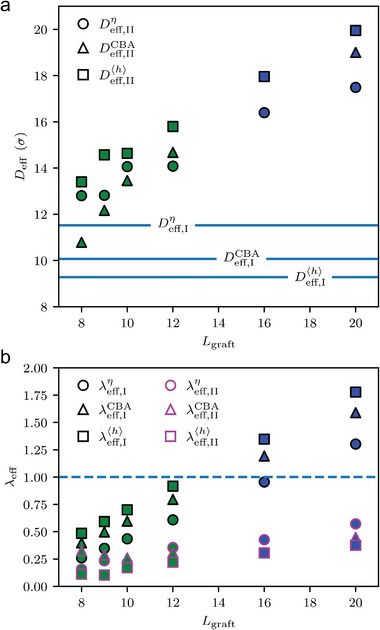
The *D*_eff_ and λ_eff_ are plotted as a function of *L*_graft_. As discussed in the section on polymer conformation, two types of transitions can be observed. a) These transitions are labeled as type I and II, with type I characterized by *D*_eff, I_ that is invariant to chain length, and type II characterized by *D*_eff, II_ that scales with chain length. The green (close‐packed structure) and blue (bcc) face colors in the figure indicate the dominant symmetry that emerges in the simulation system for a given chain length. Additionally, the dashed line in b) indicates a value of λ_eff_ = 1, around which our previous experiments^[^ ^24^, ^25^ ^]^ observed a symmetry transition. Black and magenta color edges correspond to types I and II, respectively.

We aim to address the only remaining question of how we can predict the symmetry of a superlattice composed of a given GNP using the conformation characterization that we calculated. Given that 〈*h*〉 can be expressed as a function of *s*, we can easily compute different types of effective core diameter ($D_{\mathrm{eff},\alpha }^{\beta }$) and effective softness ($\lambda_{\mathrm{eff},\alpha }^{\beta }$) by using the formulas $D_{\mathrm{eff},\alpha }^{\beta }=2\left\langle h\right\rangle \left(s_{c,\alpha }^{\beta }\right)+D$ and $L_{\mathrm{eff},\alpha }^{\beta }=L_{\mathrm{graft}}-s_{c,\alpha }^{\beta }\sigma$, respectively. In the earlier section, we calculated six types of $s_{c,\alpha }^{\beta }$, and since 〈*h*〉 was already known as a function of *s*, it was straightforward to calculate $\lambda_{\mathrm{eff},\alpha }^{\beta }=L_{\mathrm{eff},\alpha }^{\beta }\;/D_{\mathrm{eff},\alpha }^{\beta }$. The resulting values for $D_{\mathrm{eff},\alpha }^{\beta }$ and $\lambda_{\mathrm{eff},\alpha }^{\beta }$ are presented in **Figure** **7**a,b, respectively. Based on our previous experimental reports,^[^ ^24^, ^25^ ^]^ we know that the threshold between a close‐packed structure and a bcc structure is around λ_eff_ ≈ 1. In the case of type II transitions, even the longest chain shows an effective softness equal to or less than 0.5 for all three types of transitions (CBA, η, and 〈*h*〉), indicating poor consistency with the experiment. On the other hand, the type I transition accurately captures the transition around the effective softness of unity. Therefore, our findings support the hypothesis that a characteristic monomer index, which is independent of the length of the grafted polymer, is a crucial parameter that determines the *D*_eff_. This *D*_eff_, in turn, governs the effective softness and the symmetry of the GNP superlattice. Additionally, our findings do not contradict the theoretical prediction by Ohno et al.,^[^ ^39^ ^]^ which posits that the *D*_eff_ has no dependence on the length of the grafted polymer. Notably, all three types of grafted polymer conformation analyses agree well with the experimental data, indicating that the conformation characterization method can be selected relatively freely. It should be noted that type II transitions may serve as potential indicators for the transition from SDPB to mushroom conformation. This is plausible because, in the case of a flat surface, the conformational transition is known to occur at $\sigma_{g,c}=1/R_{g}^{2}$ where σ_g,c_ represents the grafting density at which conformational transitions occur, and *R*_g_ is the radius of gyration. Let us assume a height (*x*_II_) from GNP surface and consider grafts on the fictitious sphere surface of diameter $D+2x_{\text{II}}$. At sufficiently large $x_{\text{II}}\left(D+2x_{\text{II}}\to \infty \right)$, the spherical surface can be approximated as flat surface. Then the conformational transition can be specified using $\sigma_{g,c}=N_{\mathrm{graft}}/{\left(D+2x_{\mathrm{II}}\right)}^{2}\pi \;\approx 1/R_{g}^{2}$. Since $R_{g}∼L_{\mathrm{graft}}^{\nu }$, $x_{\text{II}}$ is proportional to $L_{\mathrm{graft}}^{\nu }$. Therefore, the SDPB‐to‐mushroom transition involves a relevant length scale, which is a function $L_{\text{graft}}$. Presumably, this would be represented by the type II transition points; however, this is beyond the scope of the current study and will not be discussed further.

## 4 Conclusion

In this study, our primary focus was on investigating the self‐assembly of various designs of GNP superlattices in terms of their effective softness, building on our previous works^[^ ^24^, ^25^ ^]^ in this area. Our research was guided by three key questions that have arisen from past studies of monodisperse GNPs. First, we sought to determine whether there are any unique conformational transitions available for a given GNP. Second, we investigated whether GNPs with polymers explicitly grafted onto their surface could develop into a specific symmetry starting from a random initial configuration, and whether they exhibited similar symmetry transitions to those observed experimentally. Furthermore, during these investigations, we observed that the transition from CPB to SDPB is characterized by a gradual crossover, refining our understanding of the conformational behavior of grafted polymers. Finally, our objective was to determine whether the characterized transitions could be employed to predict the symmetry and symmetry transition of GNPs. To address the research questions, we performed molecular dynamics simulations on multiple GNPs. By initiating the simulations with randomly seeded GNPs, we observed the spontaneous formation of superlattices exhibiting specific symmetries (close‐packed and bcc). These structures emerged in close proximity to the non‐ergodic boundary and were contingent upon the length of the grafted polymers. Through this approach, we were able to find six characteristic monomer indices ($s_{c,\alpha }^{\beta }$) using different characterization methods (β; CBA, η, and 〈*h*〉), and grafted polymer length dependency (α; type I and II). Furthermore, we successfully obtained self‐assembly with both close‐packed and bcc structures identified using i‐CNA^[^ ^49^ ^]^ and VoroTop.^[^ ^50^ ^]^ Ultimately, this study establishes a connection between the conformation of grafted polymers and the symmetry of GNP self‐assembly by demonstrating the predictive power of $\lambda_{\mathrm{eff},\alpha }^{\beta }$ which can be calculated using $s_{c,\alpha }^{\beta }$. This research successfully relates the fragmented concepts of polymer conformation and self‐assembly structure. By comparing the effective softness at which the experiment^[^ ^24^, ^25^ ^]^ is expected to exhibit symmetry transitions, we observe the same transitions with type I, while type II transitions suggest further investigation into the SDPB‐to‐mushroom transition, which is beyond the scope of this study and will be addressed in future work. Overall, this research contributes to the quantitative determination of effective softness and symmetry prediction by delineating the boundaries separating two distinct regions (CPB and SDPB) of the grafted nanoparticle through conformation transitions. This enhances our understanding of grafted polymer physics in conjunction with macroscopic symmetry of GNP superlattices. In our current study, while the effect of grafting density has not been the primary focus due to our aim for conciseness, its influence remains a significant motivator for our work and will be comprehensively addressed in our future research. Additionally, the investigation of geometric confinement, particularly the body‐centered tetragonal (bct) symmetry identified in our earlier work but not explored in this study, will be a key area of focus in our forthcoming endeavors. This future work aims to build upon the foundational findings of our current study, exploring these critical aspects in greater depth.

## 5 Experimental Section

### GNP Design

GNP is designed as a composite body which is similar to the one used in the work of Midya et al.^[^ ^41^ ^]^ but the grafting points are chosen to minimize the surface area of the convex hull formed by the points. The core of GNP and the monomers at grafting points are constrained as a rigid body. The key design parameters are the number of grafted polymers (*N*_graft_) and the degree of polymerizations (*L*_graft_). For the grafted polymer chains, Kremer‐Grest bead‐spring model is used.^[^ ^54^, ^55^ ^]^ FENE bond (Equation 1) is used for all bonds and WCA potential (Equation 2) is used for interaction between a pair of monomers separated by a distance within pair interaction cutoff *r*_c_ = 2^1/6^σ where σ is Lennard‐Jones (LJ) length unit. The WCA Interaction parameter is ε_*ij*_ = 1.0ε where ε is LJ unit energy.

(1) $$U_{\mathrm{FENE}}\;\left(r_{ij}\right)=-\frac{1}{2}k_{\mathrm{bond}}r_{0}^{2}\ln\left(1-{\left(\frac{r_{ij}}{r_{0}}\right)}^{2}\right)$$

(2) $$U_{\mathrm{WCA}}\;\left(r_{ij}\right)=4\varepsilon_{ij}\;\left({\left(\frac{\sigma }{r_{ij}}\right)}^{12}-{\left(\frac{\sigma }{r_{ij}}\right)}^{6}+\frac{1}{4}\right)$$

For core–core and core–monomer interactions, the expanded WCA potential (Equation 3) is used to take the size effect into account.

(3) $$U_{\mathrm{shifted}}\;\left(r_{ij},\;\Delta_{ij}\right)=4\varepsilon_{ij}\;\left({\left(\frac{\sigma }{r_{ij}-\Delta_{ij}}\right)}^{12}-{\left(\frac{\sigma }{r_{ij}-\Delta_{ij}}\right)}^{6}+\frac{1}{4}\right)$$

The size parameter is defined as Δ_*ij*_ = (*d_i_* + *d_j_*)/2 − 1, and *d*_monomer_ = 1.0 for monomer and *d*_core_ = *D* for GNP core where *D* is the diameter of core, respectively. In all cases, the grafting density is fixed to $\Sigma =N_{\text{graft}}/\pi D^{2}=2.83/\sigma^{2}$.

### Self‐Assembly Procedure

GNPs are randomly seeded into a very large simulation cell of which the length of each dimension is $L_{\text{box}}={\left[(D^{3}N_{\text{GNP}}+N_{\text{graft}}L_{\text{graft}}\sigma^{3})\pi /6\Phi \right]}^{1/3}$ where *N*_GNP_ is the number of GNPs and $\Phi =10^{-5}$ is the volume fraction of particles for very dilute initial simulation box. The total number of particles (*N*) within the simulation box is given by *N* = (1 + *N*_graft_ *L*_graft_) *N*_GNP_. The largest system tested has N reaching up to 1080250 (*D* = 4.5σ, *N*_graft_ = 180, *L*_graft_ = 24, and *N*_GNP_ = 250). It is important to note that *N*_GNP_ is fixed at 250 for all systems to ensure minimal impact due to the difference in cubic box commensurability between the two symmetries of focus (bcc and close‐packed structures). For *l* repetitions of the unit cell in all 3D directions, there are 2*l* ^3^ particles for bcc and 4*l* ^3^ particles for close‐packed structures. Consequently, *N*_GNP_ = 250 = 2 × 5^3^ was selected as it is closely approximated by *N*_GNP_ = 256 = 4 × 4^3^. The two‐step relaxation is done before the simulation to relax the system energy. More specifically, simulation is run with soft Lennard‐Jones potential^[^ ^56^ ^]^ to pump out initial high energy, followed by additional relaxation at desired potential. The simulation is the iteration of compression, relaxation, and production in different Isothermal‐isobaric ensemble (NPT). In the compression stage of *i*‐th iteration, the target pressure is set to *P*_*i* + 1_ while the last pressure is *P_i_* except that *P_i_* = *P*_*i*+1_ in the first stage (*i* = 0). In the relaxation and production stages of the same iteration, the pressure is kept in *P*_*i*+1_. The data production is only done in production stage. The timestep is $0.005-0.01\tau (=0.01\sqrt{m\sigma^{2}/\varepsilon })$, and each compression, relaxation, production stages take 10^5^τ, 5 × 10^4^τ, and 5 × 10^4^τ, respectively, and *m* is the LJ mass unit. Throughout all iterations, the temperature is maintained at 1.0ε/*k*_B_ where *k*_B_ is the Boltzmann constant and is set to a unity. There are a total of 5 iterations at which the corresponding target pressures are *P*_1_ (= *P*_0_) = 10^−4^ ε/σ^3^, *P*_2_ = 10^−3.375^ ε/σ^3^, *P*_3_ = 10^−2.75^ ε/σ^3^, *P*_4_ = 10^−2.125^ ε/σ^3^, *P*_5_ = 10^−1.5^ ε/σ^3^, respectively. For all simulations, LAMMPS^[^ ^57^ ^]^ was used to perform coarse‐grain molecular dynamics simulation.

### Per‐Particle Symmetry and Convex Hull Analysis

To identify the local structure of each particle in the system, i‐CNA^[^ ^49^ ^]^ and VoroTop^[^ ^50^ ^]^ are used. Each particle is classified as one of disordered, bcc, and close‐packed structure (fcc or hcp) at each timestep. The particles get structure tag which is statistically most frequent structure identity throughout the production stage at each pressure condition. Above methods are used as implemented in OVITO.^[^ ^58^ ^]^

### Conformational Order Parameters

To investigate the conformation, three types of order parameters were investigated that are related to the conformation of grafted polymers. Each monomer in the *j*‐th grafted polymer was given an index *s^j^*,*j* ∈ [1, *N*_graft_] where *s^j^* can range from a member of rigid body to monomer at free end. Note that *s* without superscript simply implies any monomer index along the chain, and the computations are performed in the same way for all chains.

First, CBA was investigated for *s* > 1 as defined in Equation (4).

(4) $$\cos\theta_{s-1,s,s+1}=\frac{{\vec{b}}_{s-1,s}\cdot {\vec{b}}_{s,s+1}}{\left|{\vec{b}}_{s-1,s}\right|\left|{\vec{b}}_{s,s+1}\right|}$$

here, ${\vec{b}}_{s,s+1}$ indicates the bond displacement vector from *s*‐th monomer to (*s*+1)‐th monomer. The first (Equation 5) and the second (Equation 6) derivatives of all associated conformational order parameters with respect to the monomer index were computed as follows:

(5) $${\left.\frac{d\left(\cdot \right)}{\mathrm{dx}}\right|}_{s+0.5}\overset{\text{def}}{=}\frac{{\left(\cdot \right)}_{s+1}-{\left(\cdot \right)}_{s}}{s+1-s}={\left(\cdot \right)}_{s+1}-{\left(\cdot \right)}_{s}$$

(6) $${\left.\frac{d^{2}\left(\cdot \right)}{ds^{2}}\right|}_{s}\overset{\text{def}}{=}\frac{\frac{{\left(\cdot \right)}_{s+1}-{\left(\cdot \right)}_{s}}{s+1-s}-\frac{{\left(\cdot \right)}_{s}-{\left(\cdot \right)}_{s-1}}{s-\left(s-1\right)}}{s+0.5-\left(s-0.5\right)}={\left(\cdot \right)}_{s+1}-2{\left(\cdot \right)}_{s}-{\left(\cdot \right)}_{s-1}$$

The closed space formed by segments of grafted polymers was investigated as the second step of our analysis. Each segment included the first monomer (index 1) that is part of the rigid body. For each grafted polymer, a segment was selected, resulting in a total of *N*_graft_ segments for a single GNP. The set of monomers was determined to include in each segment by selecting all monomers whose index *s^j^* was less than or equal to *k*. Next, the resulting set of point coordinates ($R^{3kN_{\mathrm{graft}}}$) was used to construct the convex hull ($S^{2}$) using the alpha‐shape method.^[^ ^46^ ^]^ The probe radius was set to 4.0σ without smoothing, and the resulting surface area (*A*) and volume (*V*) were calculated, which were then averaged over time frames and all GNPs. the order parameter was defined, sphericity (η(*k*)), up to a monomer index *k* as following:

(7) $$\eta \left(k\right)\overset{\text{def}}{=}\frac{V/A}{V_{\text{Sphere}}/A_{\text{Sphere}}}≅\frac{3V}{A\left(\langle h\rangle +D/2\right)}$$

here, the *V*_Sphere_(*k*) and *A*_Sphere_(*k*) represent the volume and surface area of a sphere whose radius is the average magnitude of the displacement vector from the center of mass to monomers with index *k*.

Lastly, the brush height (*h*) to the monomer index *s* of a grafted polymer is defined as the following equation.

(8) $$h\left(s\right)\overset{\mathrm{def}}{=}\left|{\vec{r}}_{\mathrm{core}}-\vec{r}\left(s\right)\right|-\frac{D}{2}$$

where ${\vec{r}}_{\mathrm{core}}$ is the coordinates of the GNP core to which the grafted polymer belongs.

## Conflict of Interest

The authors declare no conflict of interest.

## Supporting information

Supporting Information

Supplemental Movie 1

## Data Availability

The data that support the findings of this study are available from the corresponding author upon reasonable request.
