# Supplementary material for: Two‐Regime Conformation of Grafted Polymer on Nanoparticle Determines Symmetry of Nanoparticle Self‐Assembly
Source: Adv Sci (Weinh). 2024 Jul 29;11(36):2406720. doi: 10.1002/advs.202406720 (PMC11422811; doi:10.1002/advs.202406720)

**Supporting Information**

**Two-Regime Conformation of Grafted Polymer on Nanoparticle Determines Symmetry of Nanoparticle Self-assembly**

*Ji Woong Yu^1^, Hongseok Yun*, Won Bo Lee*, YongJoo Kim**

Ji Woong Yu

Center for AI and Natural Sciences, Korea Institute for Advanced Study, Seoul 02455, Republic of Korea

Hongseok Yun

Department of Chemistry and Research Institute for Convergence of Basic Science, Hanyang University, Seoul 04763, Republic of Korea

E-mail: yunhs@hanyang.ac.kr

Won Bo Lee

School of Chemical and Biological Engineering, Institute of Chemical Processes, Seoul National University, Seoul, 08826 Korea

E-mail: wblee@snu.ac.kr

YongJoo Kim

Department of Materials Science and Engineering, Korea University, Seoul, Republic of Korea

Email: cjyjee@korea.ac.kr


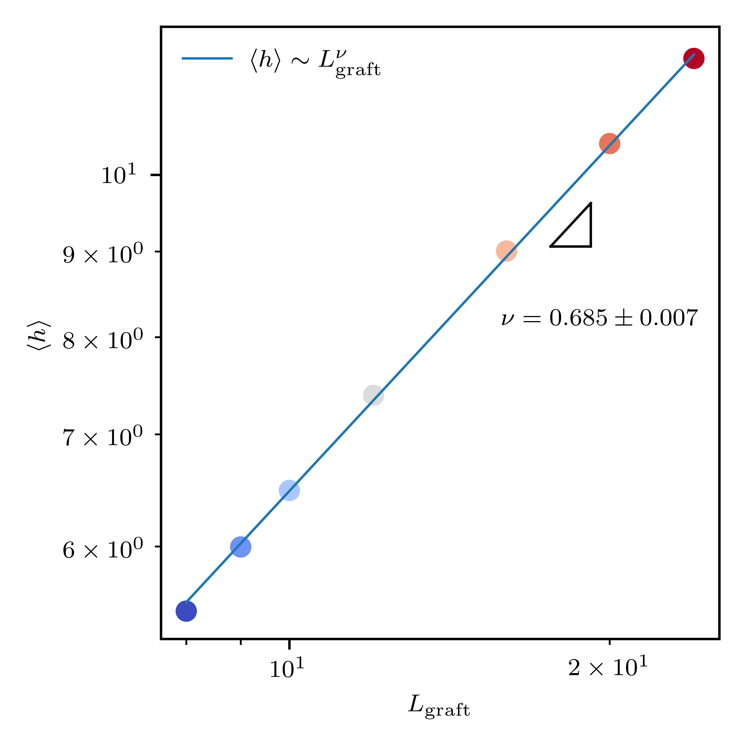


**Figure S1**. The scaling of $\left\langle h \right\rangle$ as a function of $L_{\mathrm{graft}}$. The scaling exponent, $\nu$, of $\left\langle h \right\rangle\sim L_{\mathrm{graft}}^{\nu}$ is $\nu=0.685\pm0.007$.


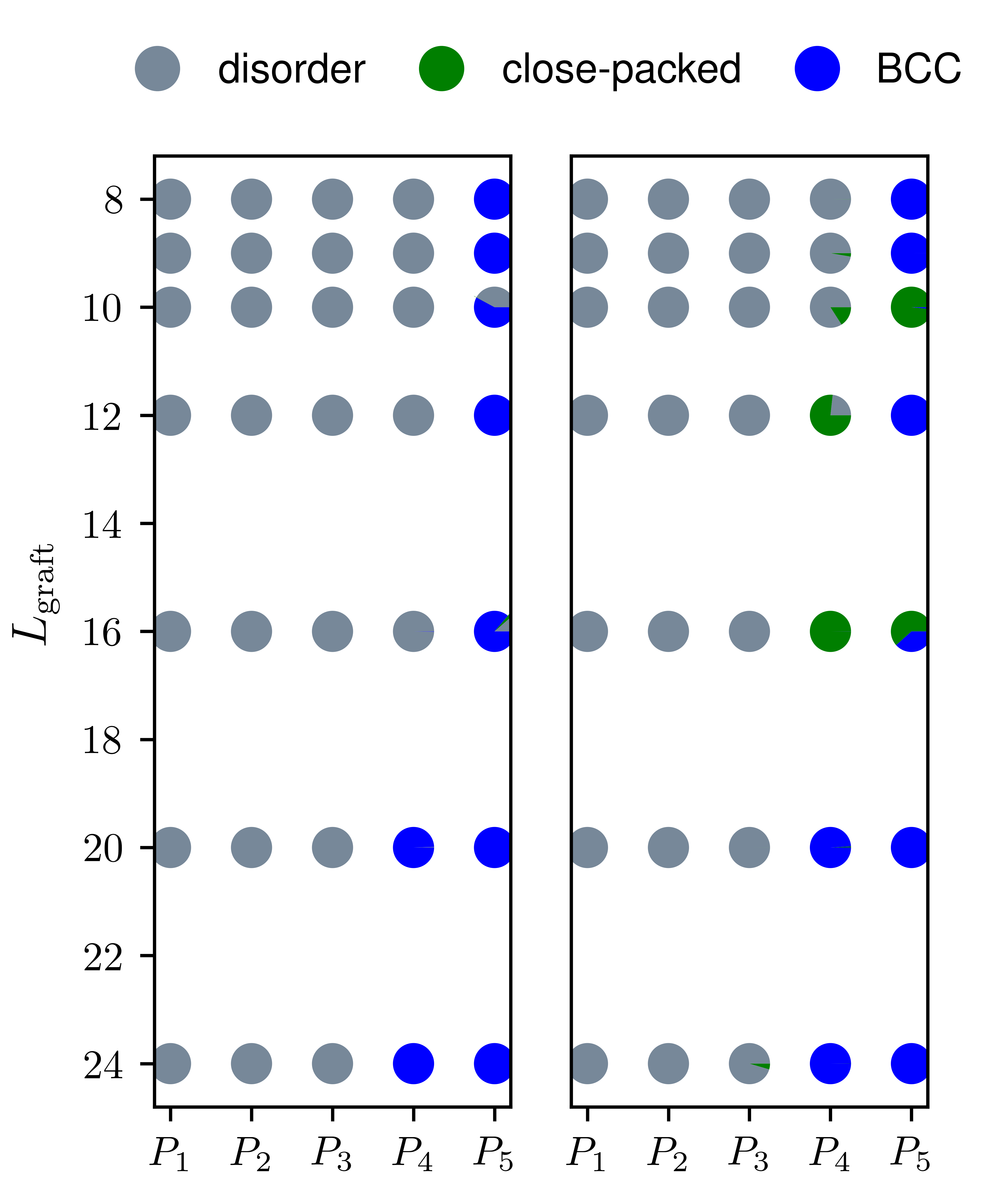


**Figure S2**. The phase diagram of grafted nanoparticles with diameter, $D=2.25$


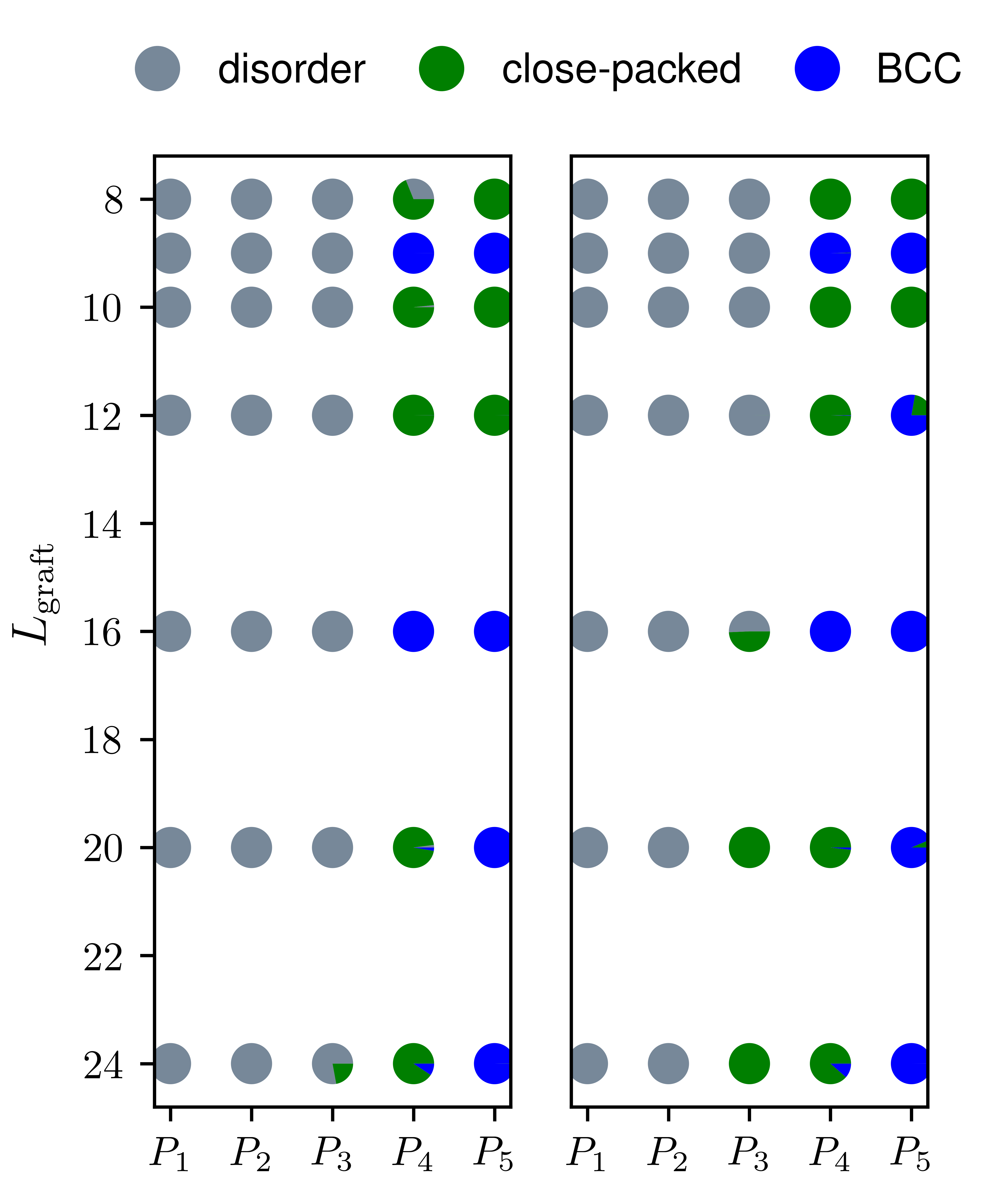
**Figure S3**. The phase diagram of grafted nanoparticles with diameter, $D=3.00$

**Figure S4**. The phase diagram of grafted nanoparticles with diameter, $D=4.50$


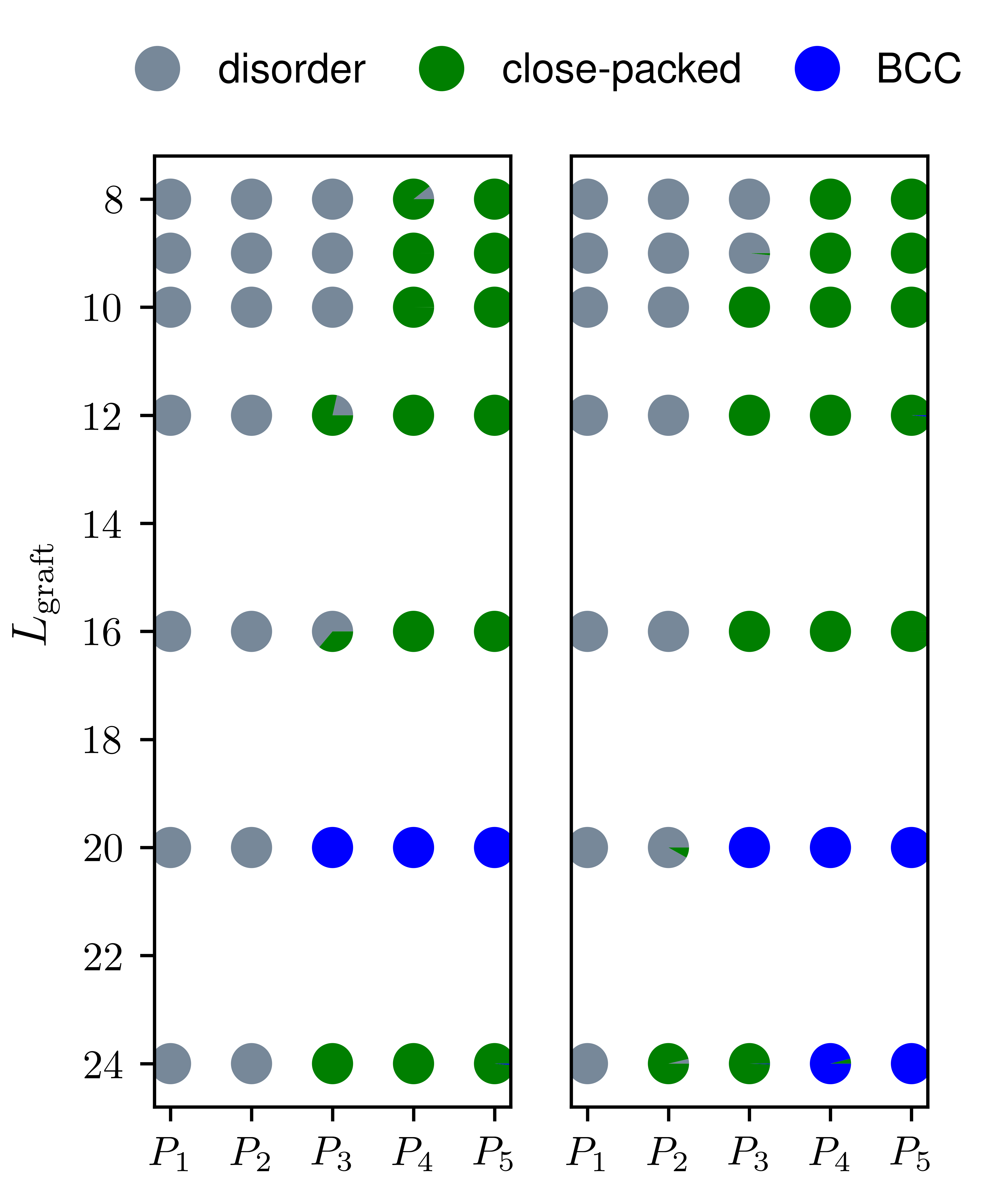

Supplement: Supplementary file 1 — Supporting Information [file ADVS-11-2406720-s002.docx]
